# Knowledge, Attitude and Practice of Research Ethics among Researchers in Nepal

**DOI:** 10.31729/jnma.8492

**Published:** 2024-03-31

**Authors:** Namita Ghimire, Santoshi Adhikari, Subhanshi Sharma, Rojina Basnet, Richa Acharya, Shashi Verma, Pradip Gyanwali

**Affiliations:** 1 Nepal Health Research Council, Ramshah Path, Kathmandu, Nepal

**Keywords:** *ethics*, *health*, *Nepal*, *research*, *researchers*

## Abstract

**Introduction:**

The universal health research ethical principles must be adhered to ensure a balance between science and safeguarding participants' rights, safety and dignity. A cross-sectional study was conducted to assess the knowledge, attitude, and practice of research ethics among researchers in Nepal.

**Methods:**

The study was carried out among 449 researchers who submitted proposals for ethical review and approval from the Ethical Review Board of the Nepal Health Research Council between January 2017 to August 2021. Simple random sampling was done ensuring a proportional representation of researchers from all areas of health research. A structured questionnaire was administered online for data collection.

**Results:**

The participants aged between 23-80 years old consented to complete the survey questionnaire. The median age of the respondents was 35 (23-80) years. Among all the respondents, 52 (11.58%) were unaware about the National Ethical Guideline for Health Research. Similarly, 110 (24.50%) respondents strongly agreed that the ethical review process impairs research and makes it harder for researchers to conduct research; 372 (82.85%) respondents had pursued research activity only after obtaining ethical approval.

**Conclusions:**

Half of the respondents had knowledge on different aspects of research ethics.

## INTRODUCTION

Health research involving human participants must be conducted adhering to ethical principles, and national and international codes of conduct.^1,2^ Proper understanding of ethics is necessary to address ethical dilemmas and adopt measures to avoid any form of intended or unintended exploitation or coercion.^3^

With the advancement in the area of health research, various guidelines and regulations have been developed accordingly for the ethical conduct of research. However, awareness and knowledge regarding research ethics among researchers is an area of concern.^4^ A comprehensive understanding of investigators' consideration of ethical aspects during health research could assist in identifying relevant training gaps and provide further impetus to policymakers to strengthen ethical review systems. Familiarizing researchers with research ethics and ethical guidelines also aids in enabling participation in international research collaborations.^5^

This study was conducted to assess the extent to which investigators are cognizant of ethical considerations in research and to identify gaps in knowledge and practice of research ethics.

## METHODS

A descriptive cross-sectional study was conducted among the researchers in Nepal who had applied proposals for ethical review and approval at the Ethical Review Board of the Nepal Health Research Council (NHRC). Sample size was calculated using the formula:


$$\begin{array}{ll} \text{n} & ={\text{Z}}^{2}\times \frac{\text{p}\times \text{q}}{{\text{e}}^{\text{2}}}\\ & =1.96^{2}\times \frac{0.50\times 0.50}{0.05^{2}}\\ & =385 \end{array}$$

Where,

n = minimum required sample size
Z = 1.96 at 95% Confidence Interval (CI)
p = prevalence taken as 50% for maximum sample size
q = 1-p
e = margin of error, 5%

The calculated minimum required sample size was 385. Adding 15% non-response rate, the final sample size was 443. Research projects in Nepal related to health must receive ethical approval from the Ethical Review Board of the NHRC. The study is based on the proposals received from January 2017 to August 2021 in 30 different areas of research as specified by the NHRC. The proposals that were rejected and withdrawn were excluded from the study. Samples were selected using proportional random sampling. Further, participants from each area of research were selected using simple random sampling to generate a random number table.

Researchers' email addresses were sourced from the ERB's online database with administrative approval from the Nepal Health Research Council. Ethical approval from the Ethical Review Board (Reference number: 680/2021) was obtained prior to the initiation of the study. Pretesting of the questionnaire was also done. The information sheet along with the informed consent form was shared with the researchers through Google Docs. The information sheet thoroughly outlined the components of the research, and adequate information was provided. Participants could indicate their consent to proceed with the research by marking "agree" in the Google Docs. The questionnaire was accessible only when the participants provided consent for their participation in the study.

A structured questionnaire was developed with reference to existing literature in consultation with subject experts, taking an adequate sample size, and making the tools comprehensive.^1,4,6-10^ There were 12 questions to assess knowledge of research ethics, and 10 statements to assess attitude towards research ethics using a 5-point Likert scale (strongly agree, agree, neutral, disagree, strongly disagree). There were 9 practice related questions to assess the researcher's conduct of research ethics. Responses of "yes", "no", and "don't know" for knowledge were given the scores of 1, 2 and 3 respectively. Responses of "strongly disagree", "disagree", "neutral", "agree", and "strongly agree" for attitude were given scores of 1, 2, 3, 4, and 5, respectively, whereas responses "yes" and "no" for practice were given the scores of 1 and 0 respectively.

The obtained responses were entered in MS Excel and analyzed. Descriptive statistics of frequency and percentage were used to describe the categorical data and the mean with standard deviation (SD) for the numerical data. The obtained data were anonymized and the confidentiality of the participants was maintained throughout the study. Point estimate at 95% CI was calculated.

## RESULTS

A total of 3677 proposals were received for ethical review and approval from January, 2017 to August, 2021 in 30 different areas of research as specified by the NHRC, of which only 3601 were eligible for this study following the rejection and withdrawal of 76 proposals. Out of the 3601 eligible proposals, only 449 were respondents.

There were 244 (54.34%) female respondents. The age range was 23-80 years, with a median age of 35 (2380) years. The respondents were 118 medical doctors (26.28%), 80 (17.81%) public health professionals, and others as mentioned in (Table 1).

**Table 1:** Socio-demographic characteristics of the Respondents (n= 449).

Characteristics	Category	n (%)
Age (in years)	<30	108 (24.05)
	30-40	196 (43.65)
	40-50	102 (22.72)
	≥50	43 (9.58)
Sex	Male	205 (45.66)
	Female	244 (54.34)
Educational level	Bachelor level	78 (17.37)
	Postgraduate level	315 (70.16)
	PhD and above	56 (12.47)
Occupation	Medical doctor	118 (26.28)
	Public health professional	80 (17.81)
	Nurses	63 (14.03)
	Academician	56 (12.47)
	Researcher	37 (8.24)
	Allied Health Professional	34 (7.60)
	Student	29 (6.45)
	Others	32 (7.12)
Work experience	<1 year	34 (7.57)
	1-5 years	145 (32.30)
	>5 years	270 (60.13)
Number of publications	No publication	96 (21.39)
	1-5 publications	159 (35.41)
	>5 publications	194 (43.20)
Training in research	Yes	352 (78.40)
	No	97 (21.60)

Out of 449 respondents, 380 (84.63%) could correctly highlight the importance of research ethics. However, 52 (11.58%) were unaware of the National Ethical Guidelines for Health Research in Nepal; 421 (93.76%) respondents knew that informed consent is required from research participants before starting a study; 247 (55.01%) respondents believed that informed consent is not required for retrospective studies. Additionally, 103 (22.94%) were unaware that Good Clinical Practice training is required for conducting clinical trials; 435 (96.88%) respondents knew the legal age for providing consent in Nepal, 99 (22.05%) respondents knew about written assent (Table 2).

**Table 2:** Knowledge regarding research ethics among researchers (n = 449).

Characteristics	Category	n (%)
What is the importance of the research ethics?	To protect the right of research participants	59 (13.14)
	To protect the welfare of the research participants	8 (1.78)
	Both of the above	380 (84.63)
	None of the above	2 (0.45)
Which of the following is/are considered guidelines in research ethics?	Declaration of Helsinki	88 (19.60)
	Nuremberg Code	24 (5.34)
	Belmont Report	7 (1.56)
	All of the above	330 (73.50)
Which of the following is/are basic ethical principles?^*^	Autonomy	289 (64.36)
	Beneficence/Non-maleficence	302 (67.26)
	Conflict of interest	153 (34.10)
	Authorship	85 (18.90)
Is there any ethical guideline for conducting health research in Nepal?	Yes	397 (88.42)
	No	12 (2.67)
	Don't Know	40 (8.91)
Informed consent is a consent given by a competent individual after getting all the necessary information regarding the research.	Yes	392 (87.31)
	No	51 (11.36)
	Don't Know	6 (1.33)
When the informed consent is taken?	Before starting the research activity	421 (93.76)
	After completion of research activity	26 (5.80)
	During the research activity	2 (0.44)
Which type of consent is required while conducting retrospective study?	Written Consent	145 (32.29)
	Verbal Consent	57 (12.70)
	Consent is not required for retrospective study	247 (55.01)
Can changes in approved research proposals be made without research ethics committee approval?	Yes	27 (6.01)
	No	370 (82.41)
	Don't Know	52 (11.58)
Training of good clinical practice is mandatory for conducting clinical trials?	Yes	346 (77.06)
	No	33 (7.35)
	Don't Know	70 (15.59)
Who should have the ownership of the research data?	Principal Investigator	394 (87.75)
	Funding Agency	28 (6.24)
	Collaborators	27 (6.01)
Written assent is required for which age group of participants?	Below 7 years	350 (77.95)
	7-18 years	99 (22.05)
What is the legal age for providing consent in Nepal?	Below 12 years	3 (0.67)
	12-18 years	11 (2.45)
	18 years and above	435 (96.88)

* multiple responses

Researchers' attitudes towards research ethics were assessed using 10 statements. Out of total participants, 373 (83.07%) strongly agreed that research ethics should be taught as a mandatory subject to postgraduate students in the curriculum, 330 (73.50%) strongly agreed that investigators should receive training in research ethics, 110 (24.50%) strongly agreed that the ethical review process impairs research and makes it harder for researchers to conduct research. Similarly, 276 (61.47%) of the respondents strongly disagreed with fabricating research data, even if it did not cause any harm to participants (Table 3).

**Table 3:** Attitude towards research ethics among researchers (n = 449).

Statement n(%)	Strongly Agree n(%)	Agree n(%)	Neutral n(%)	Disagree n(%)	Strongly Disagree n(%)
Research ethics must be taught as a mandatory subject in the curriculum of post graduate students.	373 (83.07)	69 (15.37)	2 (0.45)	1 (0.22)	4 (0.89)
All investigators must have training in research ethics.	330 (73.50)	105 (23.38)	11 (2.45)	3 (0.67)	-
Ethical review of proposals delays the research and makes it harder for the researcher.	110 (24.50)	119 (26.50)	66 (14.70)	110 (24.50)	44 (9.80)
Ethical review of research should be restricted to international research and projects only.	13 (2.89)	22 (4.89)	45 (10.02)	185 (41.20)	184 (41.0)
Patients should not be informed of full research details including risks and benefits or else they may refuse to participate in the study.	35 (7.80)	22 (4.90)	15 (3.34)	95 (21.16)	282 (62.80)
There is no need to obtain informed consent to do research on blood samples already withdrawn for clinical tests.	11 (2.45)	47 (10.47)	44 (9.80)	167 (37.20)	180 (40.08)
For vulnerable groups such as children or mentally ill, informed consent should be obtained from legally authorized representatives.	313 (69.71)	115 (25.61)	14 (3.12)	7 (1.56)	
When approaching women to participate in a study, one must always obtain informed consent from the woman's husband or another dominant male person in the family.	9 (2.00)	22 (4.90)	31 (6.90)	116 (25.84)	271 (60.46)
It is okay to fabricate data to improve the outcome of research as long as there is no harm to the patients.	10 (2.23)	37 (8.24)	46 (10.24)	80 (17.82)	276 (61.47)
When obtaining data from the individuals, measures should be kept in place to protect the data from disclosure.	265 (59.02)	146 (32.52)	26 (5.80)	6 (1.33)	6 (1.33)

A total of 372 (82.85%) respondents conducted research only after obtaining ethical approval, 431 (96.0%) obtained informed consent from participants during research (Table 4).

**Table 4:** Practice of research ethics among researchers (n = 449).

Characteristics	Category	n (%)
Pursued research before obtaining ethical approval	Yes	77 (17.15)
	No	372 (82.85)
Ever obtained informed consent from participants while conducting research	Yes	431 (96.0)
	No	18 (4.0)
Information sheet was free of scientific and technical terms (n = 431)	Yes	358 (83.06)
	No	73 (16.94)
Language used in informed consent form (n = 431)	Nepali/local language	27 (6.27)
	English	404 (93.73)
Provided adequate time to participants for reading and understanding information sheet before signing the consent form (n = 431)	Yes	419 (97.21)
	No	12 (2.79)
Used any method to assess if the participants understood the information provided in the consent form (n = 431)	Yes	364 (84.45)
	No	67 (15.55)
Maintained confidentiality and privacy while obtaining consent from the participants (n = 431)	Yes	423 (98.14)
	No	8 (1.86)
Provided a copy consent to the participants (n = 431)	Yes	218 (50.58)
	No	213 (49.42)
Shared research data with others (colleagues) before publication	Yes	106 (23.60)
	No	343 (76.40)

## DISCUSSION

This study, the first of its kind in Nepal to assess researchers' knowledge, attitudes, and practices of research ethics, found that 352 (78.40%) of respondents had received training in different areas of research. This suggests that more emphasis should be placed on educating researchers in ethical principles and human subject protection to ensure the responsible conduct of health research.

The study revealed that nearly three-quarters of the respondents demonstrated knowledge of the basic principles of research ethics. The majority of the respondents were aware of different international and national ethical guidelines for health research. This finding was similar to the study conducted in Myanmar among postgraduate students.^8^ Nearly half of the survey respondents indicated that consent is necessary while conducting a retrospective study of stored data. However, the retrospective study of stored data does not necessitate written informed consent but permission must be sought from the institution from which the data will be procured.^11^ The majority of the respondents thought that Principal Investigator should have the ownership of the research data. On the contrary, only (48%) of the faculty members from the University of Jordan thought the Principal Investigator should have ownership of the research data.^12^ The reason for this discrepancy may be due to the difference in respondents, i.e., the participants of this study were researchers, whereas the participants in the study in Jordan were faculty members. Very few participants 14 (3.12%) were unaware of the age group from whom informed assent should be obtained while conducting research on children. The result indicated that almost all of the respondents 421 (93.76%) were aware of the necessity to obtain informed consent prior to beginning research activity, which is consistent with the results of a study conducted in India (93.6%).^13^

Nearly all respondents 442 (98.44%) agreed that research ethics must be taught as a mandatory subject in the curriculum of postgraduate students. The findings of this study are in line with a similar study conducted in Jordan, Egypt,and Kerela.^12,14,15^ Almost half of the survey participants reported that the review of proposals by the ethics committee delayed the research activities, which is in accordance with the study conducted in North India.^16^ This may be due to inadequate knowledge regarding the ethical review process and delayed review due to the delayed submission of the required documents for ethical clearance. This necessitates the need for training for researchers to become more familiar with the ERB and its review process along with the required document for fast review of research proposals. In this study, a few participants 58 (12.92%) agreed that research can be done using already stored blood samples without obtaining informed consent from the participants, similar to a study in Egypt.^13^ This might be due to the belief that using the sample already collected does not cause any harm to the participants, so informed consent is not needed to use the sample. In this study, a relatively similar proportion of respondents 47 (10.47%) felt that it is acceptable to fabricate data in order to achieve desired outcomes if it does not harm the study participants, which is in line with the finding of a study conducted among professionals in dental colleges of Kerala and North India and is in contrast to the findings of a study conducted in India among the medical faculty.^15,17^ These varying attitude of respondents might be due to the varying perspectives of two different groups (a medical faculty from India and a researcher from Nepal). The study participants also included a wider range of participants from various backgrounds, who likely had different levels of understanding of research ethics.

More than one in ten respondents in this study had conducted research before obtaining ethical approval. Of those who used informed consent forms, most used simple and easy-to-understand language, but only about half provided a copy of the form to participants. This suggests that many researchers still lack adequate knowledge of the consent process, which requires education on informed consent practices and documentation.

Although the existing National Ethical Guideline for Health Research clearly outlines the principles of research ethics for promoting responsible conduct of research, strategies should be developed and implemented to enhance knowledge and awareness among researchers. The Nepal Health Research Council can play a crucial role in developing such strategies to enhance knowledge in the area of research ethics and maintain scientific integrity.

This study used an online platform, which could have led to information bias. A face-to-face interviewing format would have been preferable to reduce the risk of bias. Additionally, the study was conducted among a selected group of researchers, so the findings may not be generalizable to all researchers. Nevertheless, this study is one of the first of its kind to assess the level of knowledge, attitudes, and practices of research ethics in Nepal, providing valuable baseline information.

## CONCLUSIONS

Nepali researchers demonstrate a positive attitude towards research ethics, yet gaps exist in knowledge, awareness, and practical implementation. The study revealed prevalent positive practices, including the consistent acquisition of ethical review and approval, and obtaining informed consent. Additionally, a majority of respondents acknowledge the importance of research ethics. However, a significant knowledge gap was found, particularly concerning specific guidelines and requirements. Misconceptions regarding informed consent and other ethical aspects were noted, highlighting the need for further education and training.
